# Nucleolin Inhibits G4 Oligonucleotide Unwinding by Werner Helicase

**DOI:** 10.1371/journal.pone.0035229

**Published:** 2012-06-04

**Authors:** Fred E. Indig, Ivana Rybanska, Parimal Karmakar, Chakravarty Devulapalli, Haiqing Fu, France Carrier, Vilhelm A. Bohr

**Affiliations:** 1 Laboratory of Clinical Investigation, Intramural Research Program, National Institute on Aging, National Institutes of Health, Department of Health and Human Services, Baltimore, Maryland, United States of America; 2 Laboratory of Molecular Gerontology, Intramural Research Program, National Institute on Aging, National Institutes of Health, Department of Health and Human Services, Baltimore, Maryland, United States of America; 3 Department of Life Science and Biotechnology, Jadavpur University, Kolkata, India; 4 Department of Radiation Oncology, University of Maryland, Baltimore, Maryland, United States of America; 5 Laboratory of Molecular Pharmacology, Center for Cancer Research, National Cancer Institute, Bethesda, Maryland, United States of America; University Medical Center Hamburg-Eppendorf, Germany

## Abstract

**Background:**

The Werner protein (WRNp), a member of the RecQ helicase family, is strongly associated with the nucleolus, as is nucleolin (NCL), an important nucleolar constituent protein. Both WRNp and NCL respond to the effects of DNA damaging agents. Therefore, we have investigated if these nuclear proteins interact and if this interaction has a possible functional significance in DNA damage repair.

**Methodology/Principal Findings:**

Here we report that WRNp interacts with the RNA-binding protein, NCL, based on immunoprecipitation, immunofluorescent co-localization in live and fixed cells, and direct binding of purified WRNp to nucleolin. We also map the binding region to the C-terminal domains of both proteins. Furthermore, treatment of U2OS cells with 15 µM of the Topoisomerase I inhibitor, camptothecin, causes the dissociation of the nucleolin-Werner complex in the nucleolus, followed by partial re-association in the nucleoplasm. Other DNA damaging agents, such as hydroxyurea, Mitomycin C, and aphidicolin do not have these effects. Nucleolin or its C-terminal fragment affected the helicase, but not the exonuclease activity of WRNp, by inhibiting WRN unwinding of G4 tetraplex DNA structures, as seen in activity assays and electrophoretic mobility shift assays (EMSA).

**Conclusions/Significance:**

These data suggest that nucleolin may regulate G4 DNA unwinding by WRNp, possibly in response to certain DNA damaging agents. We postulate that the NCL-WRNp complex may contain an inactive form of WRNp, which is released from the nucleolus upon DNA damage. Then, when required, WRNp is released from inhibition and can participate in the DNA repair processes.

## Introduction

The nucleolus is a nuclear domain long known to play a central role in ribosome biogenesis and RNA metabolism [1], [2], [3]. A key nucleolar protein is nucleolin, a RNA binding phosphoprotein [4], that plays a major role in nucleolar organization and function, especially ribosome genesis [5]. Nucleolin is found not only in the nucleolus, but also in the nucleus, cytoplasm and cell surface [6], [7]. It has recently become apparent that the nucleolus has other crucial functions beyond RNA genesis and manipulation [8]. Indeed, recent proteomic analyses of the nucleolus have shown that there are well over 500 nucleolar proteins, of which 12% are unconnected to nucleic acid metabolism or processing and over 30% are novel proteins of unknown function [9], [10], [11].

Recently, a new function of nucleolin has emerged, as a responder to cellular stress. Nucleolin rapidly translocates from the nucleolus to the nucleoplasm in response to heat shock [12], [13], can increase its RNA binding activity in response to UV and ionizing radiation [14], and can inhibit Nucleotide Excision Repair [15].

Nucleolin is an emerging stress response protein that also has homologous strand pairing activity and interacts with a number of DNA recombination complexes that are involved in homologous recombinational repair [12], [14], [16]. For example, nucleolin interacts with Replication Protein A (RPA) and the tumor suppressor p 53. While RPA binds single stranded DNA, p 53 regulates the DNA strand-transferase activity of Rad51 [17], [18]. Recent reports indicate that both nucleolin and WRN bind to Rad51 [16], [18]. It is thus likely that nucleolin participates in and modulates homologous recombinational repair of DNA.

Werner Syndrome protein, WRNp, is a major stress-response protein associated with human nucleoli. Werner syndrome (WS) is a rare autosomal recessive genetic disorder characterized by premature onset of aging symptoms and a higher incidence of cancer [19]. The WRN gene product is a 160 kDa protein of the RecQ DNA helicase family [20], a family of proteins involved in maintaining genomic stability [21]. Werner protein (WRNp) exhibits three enzymatic activities: 3′ to 5′ RNA and DNA helicase, ATPase, and exonuclease [22], [23], [24], [25]. WRNp has a nuclear localization signal (NLS) near the C-terminus of the protein and has been detected in both the nucleoplasm and nucleolus [26], [27]. A nucleolar targeting sequence has been found in WRNp [28]. The WRN protein forms functional complexes with several cellular proteins, some of which stimulate its helicase activity, such as RPA [29], [30] and TRF2 [31]. Nucleolin and Werner protein exhibit dynamic trafficking from the nucleolus to nuclear foci in response to DNA damage. We sought to determine if there is a physical interaction between nucleolin and WRNp and what would be the functional significance of this interaction in the context of nucleolar biology and nuclear trafficking of nucleolin and WRNp.

## Materials and Methods

### Proteins, Antibodies, Oligonucleotides and Cell Lines

The cloning and expression of GST-tagged WRN fragments, His_6_-WRN and full-length WRN has been described previously [30], [32]. GST tagged ΔN-NCL and Nucleolin fragments used were described by us [14]. RFP-WRN [33] was a kind gift of Dr. Marek Rusin, Maria Sklodowska-Curie Memorial Institute, Gliwice, Poland; GFP-NCL constructs are described below.

The following antibodies were purchased from Santa Cruz Biotechnology (Santa Cruz, CA): Rabbit anti-GST, rabbit anti-GFP mouse monoclonal anti-nucleolin (C23) antibody (MS-3), rabbit anti-nucleolin (H250), rabbit anti-WRN (H-300). Additional antibodies purchased were mouse monoclonal anti-nucleolus (Calbiochem, San Diego, CA), mouse monoclonal anti-nucleolin (MBL), rabbit anti-WRN1 (Novus, Littleton, CO), mouse anti-WRNp mAb (BD Transduction Laboratories, San Diego, CA). Horseradish peroxidase-, Cy2 and Cy3-conjugated secondary mAbs were purchased from Jackson Immunoresearch Laboratories (West Grove, PA). Alexa 488-conjugated secondary mAbs and the DNA stain 4′,6-diamidino-2-phenylindole dihydrochloride (DAPI) were purchased from Molecular Probes (Eugene, OR). Normal rabbit or mouse IgG (Sigma or Santa Cruz) was used as a negative control.

TERT-1604 (telomerase-immortalized normal fibroblasts were generously provided by Dr. Jerry W. Shay), HeLa, Saos-2, MO59K, MO59J and U2OS were grown in Dulbecco’s modified Eagle’s medium supplemented with 10% fetal bovine serum, 2 mM L-glutamine, 100 IU/ml penicillin, 100 µg/ml streptomycin, 1% vitamins and 1% amino acids (BRL-GIBCO Life Technologies, Inc). The SV40-transformed normal human cell line, GM00637D fibroblasts, WRN ^−/−^ transformed human AG11395 fibroblasts, human primary fibroblast MRC-5 and WRN ^−/−^ primary fibroblast AG03141C (all from Coriell Cell Repositories) were grown in minimum essential medium supplemented as above.

Oligonucleotides used to produce tetraplex DNA were purchased from The Midland Certified Reagent Company, Inc (Midland, Texas, USA). Radioactively labeled [γ32-P]dATP was purchased from Amersham.

### Immunoprecipition, SDS-PAGE and Immunoblot

Nuclear extracts of TERT-1604 or HeLa cells were prepared as described previously [34]. Whole cell extracts (WCE) were prepared with 5×10^6^ cells for each experimental point. The cells were washed with PBS and incubated with lysis buffer containing 150 mM NaCl, 50 mM Tris-HCl pH 7.5, 0.5% NP-40 and proteinase inhibitor cocktail at 4°C for 30 min. The WCE were then centrifuged at 14,000×g for 20 min. Supernatants were collected and processed for immunoprecipitation, immunoblotting and detection as described previously [35].

Care was taken to minimize the presence of nucleic acids in lysates, nuclear extracts and purified proteins. We employed salt concentration, DNA binding columns and addition of nuclease in order to reduce nucleic acid concentration to below detectable levels, as judged by absorbance.

### *In Vitro* Binding

ELISA assays were performed exactly as described in Indig et al., 2004, with purified WRN, GST-ΔN-NCL and GST-nucleolin fragments at 100 ng/ml. Experiments were repeated at least six times.

*In vitro* pull down assay was performed basically as described in [31], [36]. GST-WRN fragments were incubated with TERT-1604 or HeLa nuclear extract, while GST-nucleolin fragments were incubated with His_6_-WRN (approximately 1 µg each). Reactions were then immunoprecipitated with anti-GST, separated on 4–15% polyacrylamide gels and immunoblotted as described above. The resulting signal was visualized by chemiluminiscence (ECL Plus, Amersham Biosciences). Experiments were repeated at least three times.

### GFP-NCL Constructs and Immunoprecipitation

The full-length human nucleolin and the fragments containing amino acids 1 to 283 and 284 to 707 were cloned by PCR into the pEGFP-C3 vector (Clontech) at the Xho1/BamH1 sites. The plasmids were transiently transfected with Fugene HD at a 4∶1 ratio in 80% confluent HeLa cells. The next day, transfection efficiency was verified by fluorescence microscopy and the cells were harvested in cold PBS. Proteins were extracted in lysis buffer (150 mM NaCl, 50 mM Tris-HCl pH 7.5, 0.5% NP-40) and proteases inhibitor cocktail (Roche)) at 4°C for 30 min, centrifuged at 14000 x*g* for 20 min at 4°C. The supernatant was used for immunoprecipitation.

Immunoprecipitation was performed as described before [34]. Briefly, 2 mg of protein extracts were incubated at 4°C with WRN antibody for 1 h. Protein G coated magnetic beads were then added and the reaction was allowed to proceed over night at 4°C. The proteins were then washed in lysis buffer and twice in cold PBS before being eluted and loaded on SDS-PAGE. Western blot was performed with GFP Ab (Santa Cruz).

### DNA Damage Treatment, Indirect Immunofluorescence and Microscopy

Cells were grown on cover slips or CC2-coated slide flasks (Nunc Nalge) for 24 h and then incubated with the following DNA damaging agents for the indicated time period: Mitomycin C (0.1 µg/ml for 12 h), H_2_O_2_ 250 µM for 30 min, hydroxyurea 100 µm for 16 h, bleomycin 2.5 µg/ml for 2 h, 4NQO (0.1 µg/ml for 12 h) and CPT 15 µM for 12 h. Coverslips were processed for indirect immunofluorescence as described previously [34], except that those examined by confocal microscopy were incubated with primary antibodies for 16 h at 4°C, and secondary antibody conjugated with fluorescence dye for 1 h at room temperature. After washing three times (10 min each), the coverslips were mounted in Vectashield (Vector Laboratories) and viewed under a laser scanning confocal microscope (Zeiss 410) in separate channels (green, 488 nm; red, 561 nm). The images were then overlaid and analyzed with Metamorph imaging system 4.1 (Universal Imaging Corp.). Experiments were repeated at least three times. Approximately 30 cells were analyzed for each treatment.

### Co-Transfection and Live Cell Microscopy

U2OS cells (50 K) were plated onto MatTek dishes. After overnight growth, sub-confluent cells were transfected in serum-free medium using the Fugene 6 (Roche) reagent according to manufacturer’s instructions. The following ratio produced the best results:µg GFP-NCL:µg RFP-WRN:µl Fugene 6 1∶1.5∶18. Cells were imaged 40–66 hours post-transfection with a Zeiss 710 confocal equipped with a temperature-controlled and humidified CO_2_ chamber and with a definite focus system. Time series (2–12 hours) were obtained from cells treated with either 1 or 15 µM CPT by scanning every 30–120 seconds. Still images or movies were obtained from these series using the Zeiss Zen software. Experiments were repeated at least three times.

### Exonuclease Assay

Exonuclease assay reaction mixtures (10 µl) contained 40 mM Tris (pH 7.4), 5 mM MgCl_2_, 1 mM dithiothreitol, 0.1 mg/ml BSA, 1 mM ATP, and WRNp full-length recombinant protein (16 nM) in the presence or absence of ΔN-NCL. The amount of the double-stranded exonuclease substrate in the reaction mixture was approximately 3 fmol. Reactions were initiated by the addition of WRN protein and incubated at 37°C for 60 min. Reactions were stopped by the addition of an equal volume of formamide loading buffer (80% formamide, 0.5×Tris-borate EDTA, 0.1% bromphenol blue, and 0.1% xylene cyanol). The digestion products of these reactions were separated on 15% denaturing polyacrylamide gels, visualized using a PhosphorImager (Molecular Dynamics), and quantitated using ImageQuant software (Molecular Dynamics). Experiments were repeated at least three times.

### WRN Helicase Assay

A 34 bp forked duplex oligonucleotide [37] was used to assay WRN helicase activity in the presence of ΔN-NCL and nucleolin fragments.

Proteins and radiolabeled DNA substrates were incubated in helicase reaction buffer (50 mM Tris-HCl pH 7.5, 4 mM MgCl2, 2 mM ATP, 2 mM DTT and 0.1 mg/ml BSA) in a final volume of 20 µl. Reactions were incubated at 37°C for 20 min, then terminated by the addition of 3X stop dye (0.05 M EDTA, 40% Glycerol, 1% SDS, 0.05% bromophenol blue, and 0.05% xylene cyanol) to a final concentration of 1X. Products were resolved on a 12% native polyacrylamide gel, visualized using a PhosphoImager and quantitated using Image-Quant software (Molecular Dynamics, Palo Alto, CA). The percentage of single-stranded substrate produced by helicase activity was calculated with the following formula:

% Single-stranded = 100×*P*/(*S*+*P*).

Where *P* is the product, and *S* is the substrate. The values for *P* and *S* have been corrected after subtracting the background values in the no enzyme control. Experiments were repeated at least five times.

### UvrD Helicase Assay

The helicase reaction contained 0.5 nM^32^P-end labeled forked DNA duplex and the indicated concentration of proteins in 50 mM Tris-HCl pH 7.5, 4 mM MgCl2, 2 mM ATP, 2 mM DTT, and 0.1 mg/ml BSA. The reactions were initiated by addition of 10 fmol UvrD protein [38]. Reactions were analyzed as above. The percentage of single-stranded substrate was calculated using the same formula as in WRN helicase assay. Experiments were repeated at least three times.

### G4 Tetraplex Unwinding

G4 DNA was prepared essentially as described by Sen and Gilbert [39], but omitting the potassium salt during folding.

The G4 DNA unwinding assay was performed essentially as described by Huber and co-workers, [40]. We used the 39-mer OX1-T DNA (Oxytricha sp. telomeric) or the 49-mer TP-G4 DNA with similar results. Experiments were repeated at least six times. Phosphorimager images were contrast-enhanced using Adobe Photoshop.

### G4 Tetraplex Electrophoretic Mobility-Shift Assay (EMSA)

DNA-protein binding was assessed using EMSA, essentially as described in [36] and [31]. Experiments were repeated at least three times. Phosphorimager images were contrast-enhanced using Adobe Photoshop.

## Results

### Werner Helicase and Nucleolin Co-Precipitate

**Figure 1:**
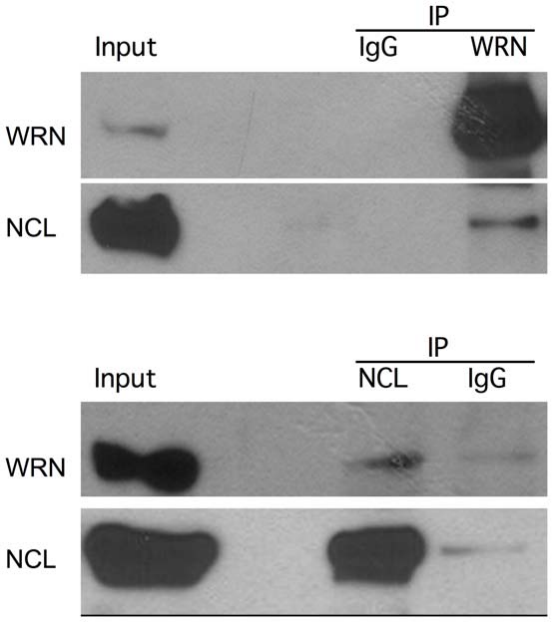
WRNp and NCL reciprocally co-immunoprecipitate. Whole cell extracts were immunoprecipitated and immunoblotted as described in Materials and Methods. Briefly, equal amounts of HeLa extract were immunoprecipitated with rabbit anti-WRN (H300, Santa Cruz, top panel), or rabbit anti-NCL (H250, Santa Cruz, lower panel). Mouse antibodies were used to detect precipitated proteins and blots were visualized with TrueBlot Western Blot kit. Control normal rabbit IgG (IgG, Santa Cruz) was used as a negative control.

Anti-WRN and anti-NCL antibodies reciprocally co-immunoprecipitate the two proteins from nuclear extracts of TERT-1604 cells (Figure 1). Similar results were obtained with other anti-NCL and anti-WRN antibodies (Figure S1A). Thus, WRNp (160 kDa) and NCL (100 kDa) are present in the same protein complex immunoprecipitated from nuclear extracts of TERT-1604 cells. The amount of co-precipitating NCL and WRN is only a fraction of the total amount of these proteins present in the extracts, as seen in Figure 1. This is unsurprising, as both NCL and WRN are multi-functional proteins that participate in several different protein complexes at the same time, and thus, only a fraction of each protein is present in each complex. When we immunoprecipitated WRNp with rabbit anti-WRN from cell extracts of six other cell lines (Figure S1B), NCL was detected in all precipitates. Both proteins were absent from anti-WRN precipitates of extracts from a WS cell line, Ag11395, producing a mutant WRN protein truncated at a.a. 369 [27], which is not precipitated by the anti-WRN.

### In Vitro Binding of WRNp and NCL and Mapping of the Interaction to the C-Termini of Both Proteins

**Figure 2:**
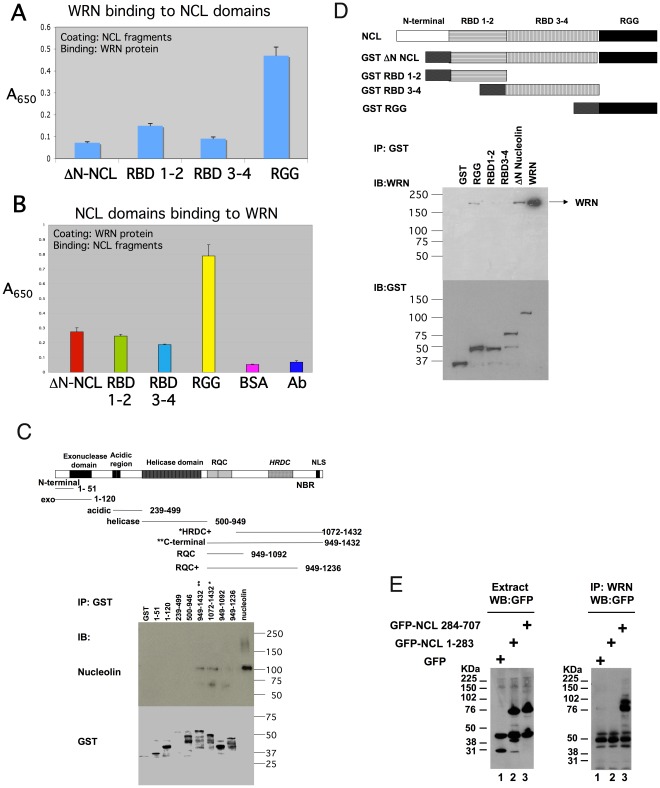
*In vitro* binding of WRNp and NCL. A-B. The WRNp binding domain of nucleolin is in the C-terminus. Indirect ELISA was performed as described in Materials and Methods. Purified GST-nucleolin fragments (A) or purified His_6_-Werner proteins (B) were coated onto 96-well microtiter plate wells. Coated protein was incubated with His_6_-Werner protein (A) or GST-nucleolin (B). Results shown were derived from a single plate, with samples analyzed in quadruplicate and error bars showing the standard deviation from the mean. The co-efficient of variation was usually less than 10%. Experiments were replicated at least three times with similar results. See section D for nucleolin fragment names and mapping. BSA, plate coated with only BSA; Ab, plate coated with only anti-WRN antibody. **C.** Different GST-WRN fragments were used to pull down nucleolin from nuclear extract as described in the Materials and Methods section. Upper panel shows the detection of nucleolin only in the WRN fragments *HRDC+(amino acid (a.a.) residues 1072–1432) and **C-terminal (a.a. residues 949–1432), but not in other fragments. NBR is the likely Nucleolin Binding Region of the Werner protein. Membrane was stripped and immunoblotted with anti-GST antibody (lower panel). MW in kDa are indicated at right for each panel. **D.** Different GST-nucleolin fragments were used to pull down full length purified His_6_-WRN. In the upper panel WRNp is present only in the nucleolin fragments containing the RGG domain- RGG and ΔN-NCL. Same membrane was stripped and immunoblotted with GST antibody (lower panel). MW in kDa are indicated at left for each panel. **E**. WRNp does not bind to NCL N-terminal domain. Constructs were transfected into HeLa cells, which were extracted and immunoblotted as detailed in Materials and Methods. (A) Western Blot analysis of Nucleolin fragments from HeLa cells transiently transfected with either pEGFP (lane 1), GFP-NCL 1–283 (N-terminal domain, lane 2) and GFP-NCL 284–707 (ΔN-NCL, lane 3). (B) Immunoprecipitation with anti-WRN antibody of the above HeLa cell extracts, and detection with anti-GFP. Lanes as above. Only in lane 3 (GFP-ΔN-NCL) is a GFP signal detected.

In order to verify the protein-protein interaction between WRNp and NCL that was indicated by the immunoprecipitation experiments, we conducted in vitro binding experiments using purified proteins (Figure 2). ELISA plates were coated with WRNp or various GST-NCL fragments (see Figure 2D). Purified WRNp preferentially bound to the immobilized RGG fragment of NCL in ELISA immunoassays (Figure 2A). When immobilized WRN protein was incubated with GST-NCL fragments, the RGG fragment was found to bind WRNp to a greater extent than the other NCL fragments (Figure 2B).

In order to map the reciprocal binding regions of NCL and WRN, we performed GST pull-down experiments. Utilizing eight GST-fused WRN fragments (Figure 2C) mixed with nuclear extract, we found that only two WRN fragments pulled down NCL: the HRDC+(WRN residues 1072–1432) and C-terminal (WRN residues 949–1432) fragments. As neither the RQC fragment (WRN residues 949–1092), nor the RQC+fragment (WRN residues 949–1236) pulled-down NCL, this result indicates that the helicase’s RQC domain is probably not involved in NCL binding. Thus, the likely NCL-interacting region of WRNp maps to WRN residues 1236–1432, which appears to constitute the main nucleolin binding region (NBR). However, as both the HRDC+and C-terminal fragments extend N-terminal to residue 1236, we cannot rule out the possibility that the WRN region 1092–1236 might also participate in this interaction.

To verify this finding, GST-NCL fragments were mixed with purified His_6_-WRN. These experiments clearly show that only the C-terminal NCL domain, present only in the RGG and ΔN-NCL fragments, binds WRNp (Figure 2D). The internal RBD 1–2 and RBD 3–4 domains were unable to pull down WRNp in this assay. To determine if WRNp interaction was through the NCL N-terminal domain, we used a third fusion system. As the very acidic NCL N-terminal end prevents efficient expression of full-length nucleolin in the bacterial expression system, we produced GFP fusion proteins in human cells. The GFP-NCL fusion constructs were expressed in HeLa cells, and cell extracts were immunoprecipitated with anti-WRN. Only the construct containing the C-terminal NCL domain, GFP-ΔN-NCL, was found to bind WRNp (Figure 2E, right panel). These data, together with the data in Figures 2A-D, indicate that the NCL N-terminal (residues 1 to 283) is not required for WRNp interaction and that the NCL RGG domain is sufficient for optimal binding.

### Camptothecin Induces Translocation of Nucleolin and WRNp from the Nucleolus to the Nucleoplasm and the Formation of Nuclear NCL-WRN Foci

**Figure 3:**
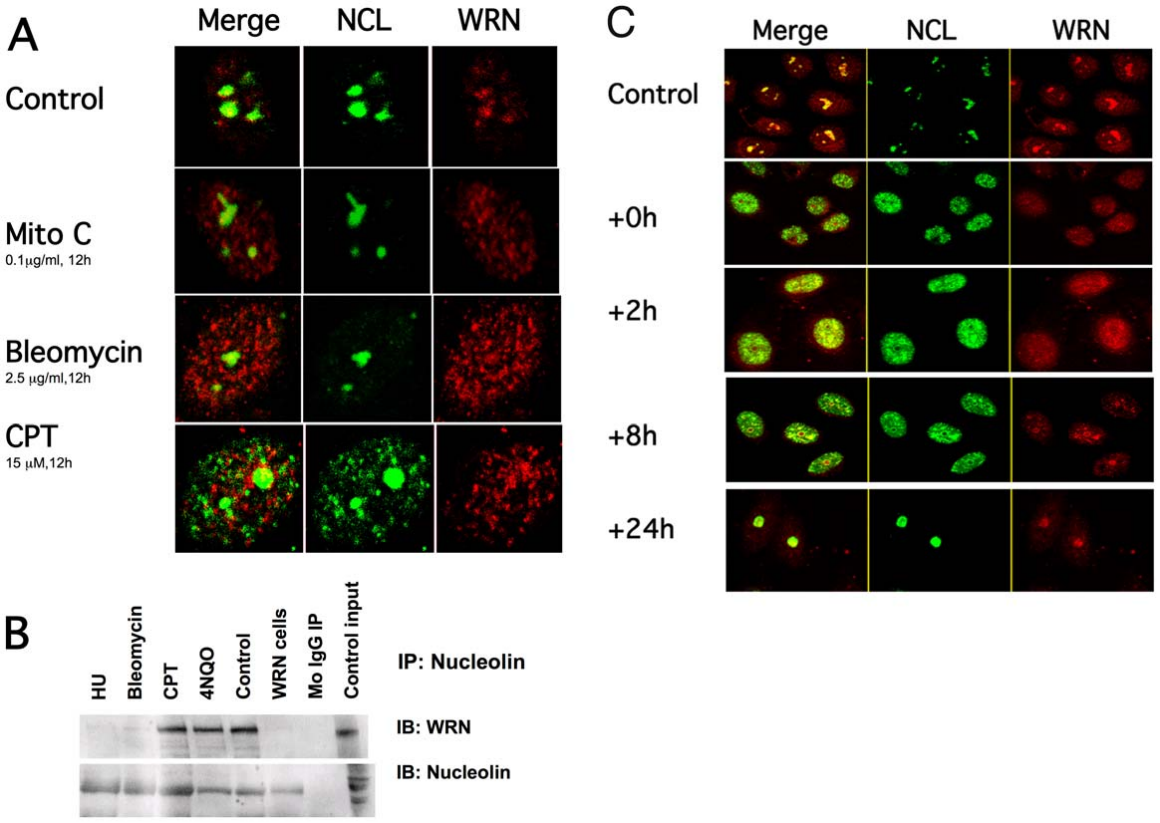
Camptothecin induces translocation of nucleolin and Werner helicase. A. Confocal microscope images of WRNp (red) and NCL (green) distribution after U2OS cells were treated with different DNA damaging agents as detailed in Materials and Methods. Fixed cells were stained simultaneously with Rabbit anti-WRN (Novus) and Mouse anti-nucleolin (Santa Cruz). Note that WRNp re-localized from the nucleolus in all treatments while nucleolin re-localized only after CPT treatment. Merged images show co-localization (yellow) of WRNp and NCL. Images are of representative cells; At least 30 cells were analyzed for each treatment, which was repeated at least three times. **B.** Cells treated as above were immunoprecipitated with anti-NCL and immunoblotted as described in the legend for Figure 1. Mito C- mitomycin C, HU- hydroxyurea, CPT- camptothecin, 4NQ0-4-nitroquinoline-1-oxide, Control- untreated U2OS (WRN plus) cells; WRN cells- Ag11395 WS cells (abnormal WRN), Mo IgG- negative control mouse IgG precipitate; Control input-10% of whole cell extract used for IP. **C.** U2OS cells were treated with 15 µM CPT for 12 h and then washed with complete medium. Cells were fixed at times from start of treatment as indicated at the left of the images, and processed for confocal microscopy as detailed in Materials and Methods.

Werner Syndrome cells (mutated Werner protein) are hypersensitive to the Topoisomerase I inhibitor, camptothecin [41], [42]. We have previously noted the remarkable effects of camptothecin on nucleolar protein complexes [34]. When U2OS cells were treated with camptothecin and several other DNA damaging agents, we found that only in the presence of camptothecin did we observe NCL (green) in the nucleoplasm (Figure 3A). Mitomycin C, bleomycin, aphidicolin (not shown) and hydrogen peroxide (not shown) did not redistribute nucleolin from the nucleolus to the nucleoplasm, although all agents caused the Werner helicase (red) to translocates from the nucleolus to the nucloplasm. Camptothecin treatment results in the formation of numerous nuclear NCL foci, some of which co-localized with WRNp foci in the nucleoplasm (Figure 3A). That only a fraction of NCL and WRN co-localize is not surprising, as both proteins interact with many other proteins and participate in several protein complexes at the same time.

Since WRNp and NCL were found to be in the same protein complex in untreated cells and nuclear extracts (Figure 1 and Figure S1), we examined the effect of DNA damaging agents on the WRNp-NCL complex (Figure 3A and 3B). Whole-cell extracts, prepared from U2OS cells that were treated with various DNA-damaging agents, were precipitated with anti-NCL. In cells treated with hydroxyurea (HU) or bleomycin only trace amounts of WRNp were precipitated with NCL. In untreated control cell lysates and in lysates from cells treated with CPT or 4NQO, more WRNp was precipitated by anti-NCL (Figure 3B). Thus, there is dissociation of immunoprecipitable WRN-NCL complexes in the cells treated with HU and Bleomycin, but not in cells treated with CPT, where some detectable WRN-NCL complexes remain. These results confirm that the effects of CPT on the WRN-NCL complex are specific and different from the other DNA damaging agents examined.

We also investigated the timing of the CPT-induced trafficking of NCL and WRNp using end-point immunofluorescence experiments (Figure 3C). U2OS cells were treated with CPT, washed and then fixed at time-points indicated. After treatment with CPT, both WRNp (red) and NCL (green) have mostly translocated from the nucleoli. At 2 hours post-treatment we observed an increase of signal intensity of co-localizing WRN/NCL (yellow) compared to the other time points, with numerous small foci containing both proteins. By 8 hours post-treatment both NCL and WRNp appear to have partially returned to the nucleoli, with several large co-localizing foci remaining in the nucloplasm. Full recovery of the pattern observed in non-treated cells (co-localizing WRNp and NCL in nucleoli) occurs between 8 and 24 hours.

**Figure 4:**
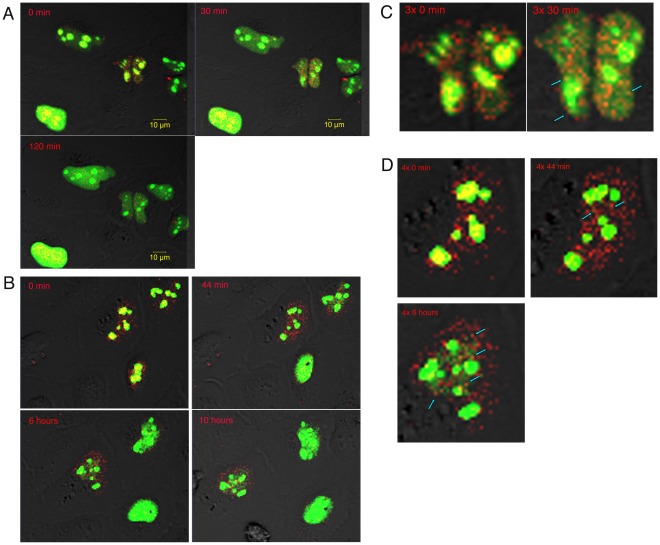
GFP-NCL and RFP-WRN co-localize in the nucleoplasm after CPT treatment. U2OS cells were transfected with GFP-NCL (green) and RFP-WRN (red) as described in Materials and Methods. Cells were treated with 15 µM CPT and immediately imaged in a time series obtained with a Zeiss 710 confocal. **A.** Still images from a 120 minute time series at 0, 30 and 120 minutes after the addition of CPT. **B.** Still images from a 14 hour time series at 0, 44 and 360 and 600 minutes after the addition of CPT. **C.** A 3x zoom on two cells from the 120 minute time series, comparing the distribution of GFP-NCL and RFP-WRN at 0 and 30 minutes. Arrows point to co-localizing WRN-NCL foci (orange-yellow) in the nucleoplasm. **D.** A 4x zoom on a cell from the overnight time series, comparing the distribution of GFP-NCL and RFP-WRN at 0, 44 and 360 minutes. Arrows point to co-localizing WRN-NCL foci (orange-yellow) in the nucleoplasm. Note the intense co-localization of NCL and WRNp in the nucleoli at 0 min, and that most of the WRNp and some of the NCL have translocated to the nucleoplasm within 30 (A) and 44 (B) minutes, where co-localizing NCL-WRN foci can be already detected (C and D).

The half-life of camptothecin is 17 minutes at these incubation conditions [43]. Thus, the original concentration of CPT is reduced to less than 2% within 2 hours. Therefore, we would expect to observe a more rapid response to CPT treatment than was observed in the indirect immunofluorescence experiments. To verify this possibility, we observed live U2OS cells that were transfected with both GFP-NCL and RFP-WRN, and then treated with 15 µM CPT (Figure 4, Movie S1). As can be seen in Figures 4A and 4B, significant amounts of RFP-WRN and GFP-NCL translocate from the nucleolus to the nucleoplasm in less than 1 hour. Furthermore, these proteins co-localize in nuclear foci after CPT treatment (Figures 4C and 4D), confirming the indirect immunofluorescence experiments. We repeated these experiments with a lower concentration of CPT, 1.0 µM (Figure S2). We observe results similar to those obtained with the higher CPT concentration of 15 µM. A dynamic proteomics analysis showed a similar rapid nucleolar reduction of certain nucleolar proteins, including nucleolin, after CPT treatment [44]. We also observed that in approximately one half of the cells expressing both GFP-NCL and RFP-WRN, nuclear foci can be clearly observed (Table S1) with both proteins co-localizing. There was a slightly higher incidence of co-localizing foci when cells were treated with 15 µM CPT compared to 1.0 µM CPT (average of 63% vs. 43%, respectively).

### Nucleolin Inhibits Werner Helicase Activity

**Figure 5:**
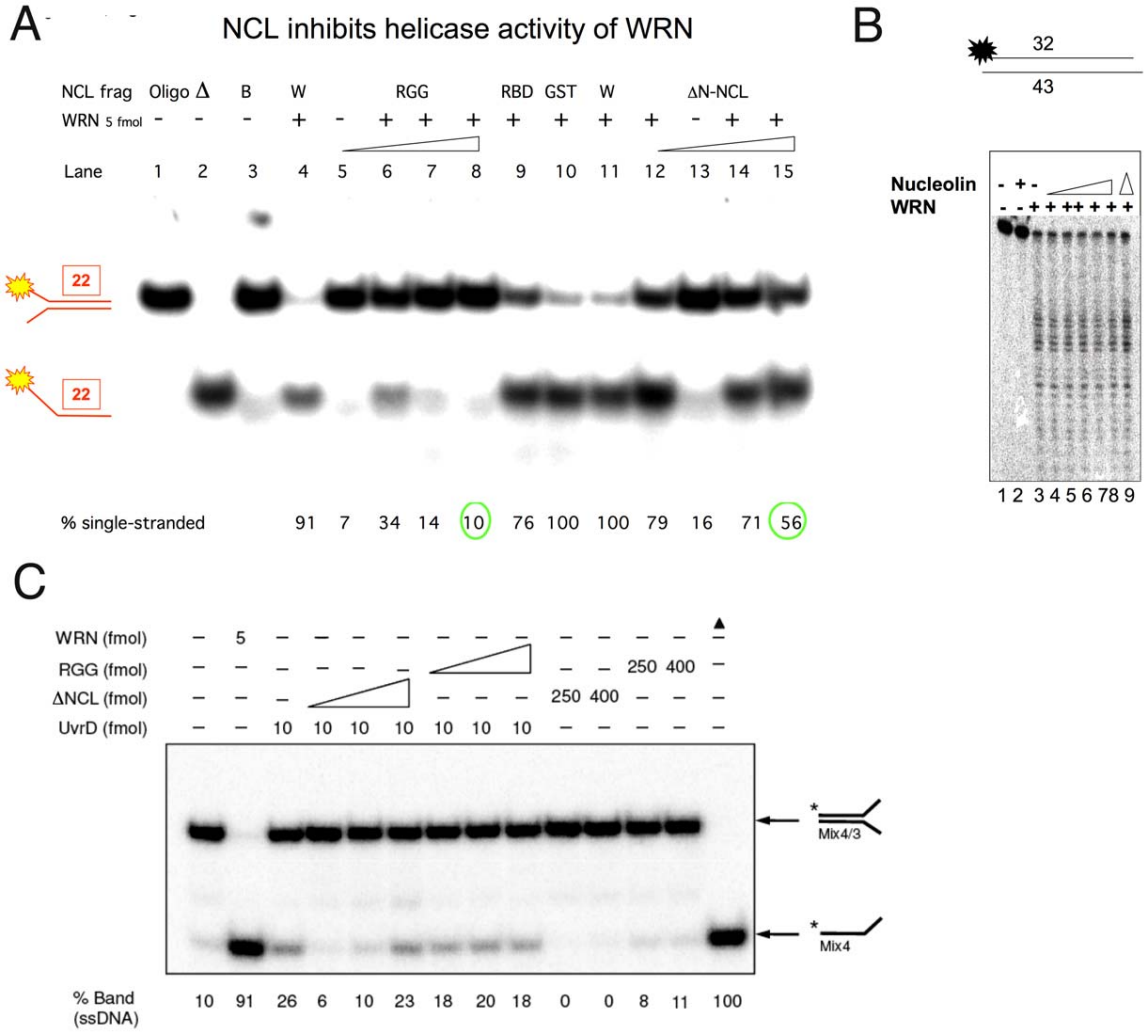
NCL inhibits WRN helicase activity but not WRN exonuclease activity. A. WRN unwinding of a helicase substrate, 22 base pair partial duplex fork substrate (shown at left), was performed as described in Materials and Methods. Purified WRNp (5 fmol) was incubated with 40 fmol substrate and 50, 125, or 200 fmol of ΔN-NCL (lanes 12–15) or RGG fragment (lanes 5–8). Controls are GST protein (200 fmol, lane 10), RBD 3–4 fragment (200 fmol, lane 9), W, only WRNp protein (lanes 4 and 11), B- only reaction buffer (lane 3), D- heat denatured substrate (lane 2), Oligo- unreacted substrate (lane 1). In Lanes 5 and 13 the WRN protein was omitted from the reaction. Green circles point out the inhibitory effect of RGG (lane 8) or ΔN-NCL (lane 15) on WRN helicase activity. **B.** WRN protein (100 fmol) was incubated with the exonuclease substrate (3′-recessed DNA substrate, represented at the top of the figure) in the presence of increasing amounts of ΔN-NCL (25, 50, 100, 200, 400 fmol) under exonuclease reaction conditions for 1 h at 37°C, as described in Materials and Methods. Once the reactions were stopped, DNA products were resolved by denaturing polyacrylamide gel electrophoresis. Controls are only reaction buffer (lane 1), only 400 fmol ΔN-NCL (lane 2), only WRN protein (lane 3) and D- heat denatured substrate (lane 9). **C.** *E. coli* UvrD protein (10 fmol) was incubated with the Mix 4/3 substrate (represented to the right of the figure) in the presence of increasing amounts of ΔN-NCL (100, 250, 400 fmol) under UvrD helicase reaction conditions for 1 h at 37°C, as described in Materials and Methods. Once the reactions were stopped, DNA products were resolved by native polyacrylamide gel electrophoresis. Controls are only reaction buffer (lane 1), WRN helicase (5 fmol, lane 2), 250 or 400 fmol ΔN-NCL without helicase (lane 10–11), RGG protein without helicase (lane 12–13) and Δ- heat denatured substrate (lane 14).

As we have established the possibility of a physical interaction between WRNp and NCL, we next examined whether NCL affected the enzymatic activity of WRNp upon known WRN substrates. The Werner protein is both a DNA helicase [22], [23] and exonuclease [24], [29] and we examined the effect of adding NCL to WRN activity assays. The helicase activity of WRN on a 22 base pair partial duplex fork substrate was efficiently inhibited by NCL. Under the conditions used, 5 fmol WRN converted 80–90% of the duplex (40 fmol) to single-strand form within 20 minutes at 37°C (Figure 5A). This conversion was inhibited by about 50% when ΔN-NCL was present at a molar ratio of 25∶1 vs WRN. The RGG fragment, which contains the putative WRNp-NCL interacting region (Figure 2), had an even greater inhibitory effect on WRNp, with over 60% inhibition of helicase activity at a 10∶1 ratio and 90% inhibition at 25∶1 (Figure 5A). Other proteins or NCL fragments, such as GST, RBD 1–2 and RBD 3–4 (not shown), had no, or only minimal (about 20%) effect on WRN unwinding of the duplex substrate at a molar ratio of 50∶1.

In contrast, we observed that NCL had no effect on WRN exonuclease activity upon a typical WRN substrate, a 3′-recessed DNA substrate (Figure 5B). To confirm the specificity of the NCL interaction with the WRN helicase, we examined a non-RecQ helicase, UvrD, for NCL effect. We found that a 40-fold molar excess of NCL or RGG over UvrD had comparatively little effect (about 20%) on UvrD activity- see Fig. 5C. A similar concentration of NCL or RGG inhibited WRN helicase activity by 2-to 4-fold (Figure 5A). As nucleolin had no effect on WRN exonuclease activity, or on a non-RecQ helicase, this indicates that NCL has a specific effect on the WRN helicase activity.

### Nucleolin Inhibits Werner Helicase Activity on a G4 Tetraplex DNA Substrate

**Figure 6:**
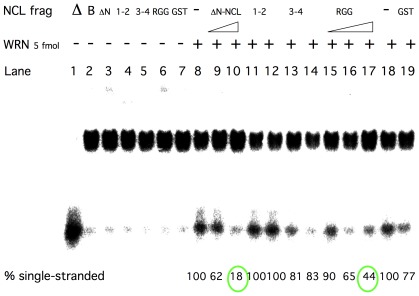
NCL inhibits WRN unwinding of G4 tetraplex DNA. The preparation of G4 tetraplex substrate was performed as described in Materials and Methods. Purified WRNp (5 fmol) was incubated with 40 fmol G4 DNA substrate and 125 or 200 fmol ΔN-NCL (lanes 9–10) or 40, 125 or 200 fmol RGG fragment (lanes 15–17). Other lanes contain controls-ΔN-only ΔN-NCL (40 fmol, lane 3), 1-1-RBD 1–2 fragment (200 fmol, lanes 4, 11and 12), 3-4-RBD 3–4 fragment (200 fmol, lanes 5, 13 and 14), GST- GST protein (200 fmol, lane 19), Only WRN protein on lanes 8 and 18, B-only reaction buffer (lane 2), Δ- heat denatured substrate (lane 1). Reactions were terminated after 20 min at 37°C and run out on 8% polyacrylamide gels. A representative intact gel is shown.

Most helicases of the RecQ family are able to unwind G4 tetraplex structures. We next sought to examine the effect of nucleolin on WRN unwinding of G4 tetraplex DNA. Under the conditions used, 5 fmol WRN converted 30–50% of the G4 DNA (40 fmol) to single-strand form within 20 minutes at 37°C (Figure 6, lanes 8 and 18). For comparison purposes, this unwinding by WRNp was defined as “100% single-stranded”, as seen in Figure 6. As with the forked duplex substrate, the presence of ΔN-NCL at a 25∶1 ratio was sufficient to inhibit 80% (lane 10) of this conversion and a similar ratio of RGG inhibited 60% of the reaction (lane 17). Again, as with the duplex fork helicase substrate, the presence of GST, RBD 1–2 and RBD 3–4 fragments had little (less than 20%) or no effect on G4 DNA unwinding by WRN.

### Nucleolin and the NCL RGG Domain Bind G4 DNA

**Figure 7:**
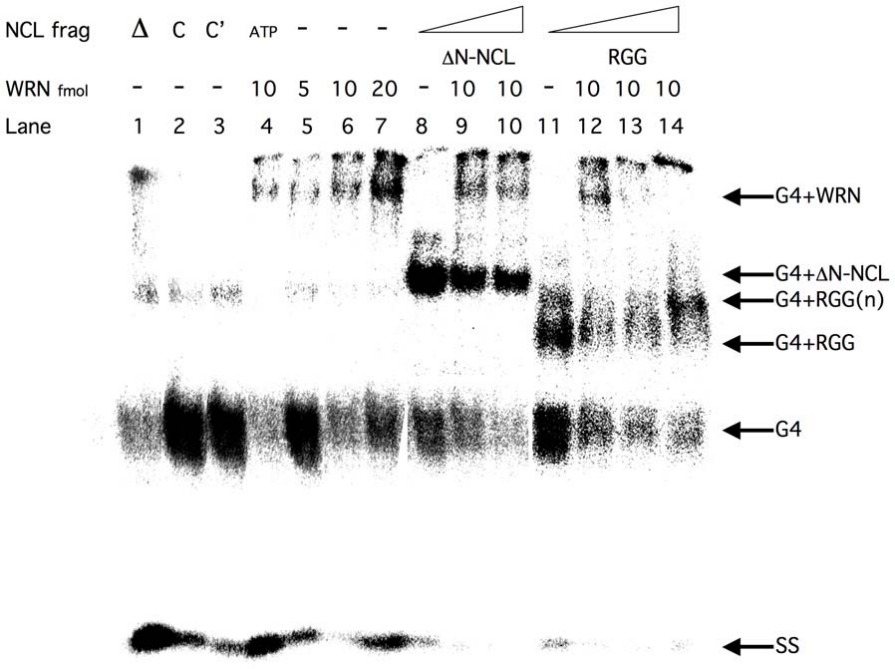
NCL competes with WRN binding of G4 tetraplex DNA. Electrophoretic mobility shift assay (EMSA) was performed for the 49-mer TP-G4 DNA with only WRNp (lanes 5–7∶5, 10, 20 fmol) and 10 fmol WRNp in the presence of ΔN-NCL (lanes 9–10∶25, 40 fmol) or RGG (lanes 12–14∶25, 40, 80 fmol). Reactions were incubated for 20 min at 37°C and run out on 5% polyacrylamide gels at a cross-linker ration of 19∶1 acrylamide:*bis*. Gels were run at 6.5 V/cm for 4–6 h at room temperature. Control lanes: Δ- heat denatured substrate (lane 1); C, C’-only reaction buffer, at 4°C (lane 2) or 37°C (lane 3); ATP-10 fmol WRNp plus ATP (all other lanes contain ATPγS); Lanes 8 and 11 contain only 25 fmol ΔN-NCL or RGG, no WRNp, respectively. The position of the oligonucleotide or oligonucleotide-protein complex is indicated at the right. A representative intact gel is shown.

Utilizing electrophoretic mobility shift assays (EMSA), we determined that WRNp, ΔN-NCL and the RGG domain could bind to G4 tetraplex DNA (Figure 7). WRNp in the presence of ATP converts the G4 form to single-strand DNA (Figure 6, lanes 8 and 18; Figure 7, lane 4). A high molecular weight (HMW) slowly-migrating G4 DNA, interpreted as a WRN/G4 complex, can also be seen in Figure 7, lane 4 and in all lanes where WRNp is present. Increasing the amount of WRNp increases the WRN/G4 DNA complex signal in a dose-responsive manner (lanes 5–7). The introduction of ΔN-NCL reduces the WRN-G4 DNA complex signal (lanes 9–10), decreases the amount of free G4 DNA present and introduces a new band, interpreted as ΔN-NCL-G4 DNA complex, that can be seen also when WRN is not present (lane 8). These data indicate that both WRNp and ΔN-NCL can bind G4 DNA when both proteins are present (lanes 9,10, 12–14) or when only WRNp (lanes 4–7) or NCL (lanes 9–10) were added to the substrate. When we add the RGG fragment instead of ΔN-NCL in the absence of WRNp, a new band, interpreted as RGG-G4 DNA complex appears (lane 11). Increasing the quantity of RGG present reduces the WRN/G4 DNA signal in a dose-responsive manner (lanes 12–14), indicating that RGG out-competes WRNp for G4 DNA. It is also possible that RGG and WRNp bind G4 DNA and supershift it, resulting in the reduced G4-WRN signal of lanes 13 and 14. Furthermore, increasing the amount of RGG leads to a shift of the RGG-G4 complex signal from a faster migrating position (lane 11) to a slower migrating one (lane 14), possibly due to the increase in the number of RGG molecules binding each G4 DNA molecule (G4-RGG(n)).

## Discussion

These studies show that nucleolin is physically associated with the Werner helicase in the nucleolus and nucleus. This conclusion is based on the specific and reciprocal co-immunoprecipitation of these proteins, in vitro binding assays and co-localization by indirect immunofluorescence in confocal optical sections, and in live cells transfected with both proteins. We have identified the C-terminal domains of both proteins as the interacting regions, and have determined that WRNp has a nucleolin binding domain, probably in the region of aa residues 1236–1432. Furthermore, treatment of cells with camptothecin causes the dissociation of both nucleolin and WRNp from nucleolar complexes, followed by their translocation to the nucleoplasm, where we find WRNp and NCL in the same protein complexes. This dynamic process of protein relocation from the nucleolus following DNA damage is clearly seen in live cells transfected with GFP-NCL and RFP-WRN. Our data further suggests that NCL and WRNp both participate in complexes that include G4 tetraplex DNA.

Nucleolin co-localizes with WRNp in the nucleoli of untreated cells. This nucleolar complex was dissociated by treatment with the DNA-damaging agent, camptothecin. CPT is a DNA topoisomerase I inhibitor that blocks topoisomerase I kinase activity [45] and causes DNA strand breaks [46], [47]. Cells and cell lines derived from Werner Syndrome patients were shown to be sensitive to the genotoxins camptothecin and 4-NQO [41], [48], [49]. We observed that other genotoxic agents, such as mitomycin C and bleomycin did not dissociate nucleolin from the nucleolus as did CPT. WRNp had increased nuclear signal after treatment with bleomycin, mitomycin C and CPT, but only CPT increases nuclear dispersion of nucleolin, while possibly retaining the WRNp-nucleolin interaction. Similarly, topoisomerase I was shown to dissociate from nucleoli after treatment with the CPT derivative, topotecan, while hydroxyurea had no effect [50]. We have previously shown that CPT dissociates nucleolar protein complexes containing WRNp and topoisomerase I [51] and also dissociates the interaction between WRNp and the AAA ATPase VCP in the nucleolus [34]. It is possible that CPT dissociated WRNp from the nucleolus, but not from nucleolin, and that WRN-NCL translocate together to the nucleoplasm. However, our immunofluorescence and fluorescent protein data (Figure 3C, Figure 4 and Figure S2) argue against this. It is more likely that the events unfold as seen in the live cell experiments- after CPT treatment, both WRNp and NCL translocate to the nucleoplasm and can subsequently form complexes seen as co-localizing foci.

In addition, we note that WRNp and NCL are co-precipitated from nuclear extracts of non-treated cells and CPT-treated U2OS cells, but not from CPT-hypersensitive Werner Syndrome cells or from hydroxyurea or bleomycin treated U2OS cells. These molecular data are consistent with the observation that CPT has a specific effect on the survival of WS cells [19] when compared to other DNA damaging agents, such as UV irradiation, hydroxyurea, bleomycin and alkylating agents [19]. These results underline the complex nuclear protein trafficking that commences after the cell is exposed to DNA damaging agents.

CPT not only increased the extra-nucleolar presence of WRNp and nucleolin, but also induced the formation of multiple nucleoplasmic foci where WRNp and nucleolin co-localize. This interaction can be detected within 30 minutes after CPT treatment and the number of nuclear complexes peaks at 2–4 hours after CPT treatment and are largely gone from the nucleus after 8–24 hours of recovery, indicating that the WRNp/NCL foci are consistent with CPT-induced damage repair complexes. We should caution that this timeline is tentative, as some variability in cell response to CPT has been observed and that the details require further investigation.

The translocation of WRNp after treatment with topoisomerase I inhibitors has been observed previously with other WRNp-associated proteins. CPT induced WRNp translocation to intranuclear repair foci that included the repair proteins Rad50 and RPA [52]. Nucleolin was found to inhibit replication in response to stress conditions by binding RPA [53]. Interestingly, heat-shock also translocates nucleolin to the nucleoplasm, where it binds RPA for about two hours after treatment [13] and inhibits DNA replication initiation [54]. WRNp also translocated from the nucleolus to the nucleoplasm after treatment with the genotoxic agent 4NQO [26] and under serum starvation [55]. Thus, certain kinds of damage (CPT-induced DNA breaks, for example), prompt the release from the nucleolus of many proteins involved in DNA repair.

G4 tetraplex structures are found in rDNA, telomeric DNA and IgG DNA, regions that have abundant nucleolin [56], [57]. These complex structures are unwound in vitro by the RecQ helicase family members BLM, WRNp and Sgs1p [40], [58], [59]. Nucleolin can also bind to G4 DNA with a k_D_ = 0.4 nM [60], and is known to promote the formation of c-MYC G4 DNA ([61]). Indeed, we can see in our results that nucleolin efficiently prevents the unwinding of G4 DNA by WRNp. WRNp probably binds G4 DNA via its RQC domain, similar to BLM and Sgs1p [62], and binds the NCL C-terminal to the HDRC region (between residues 1236–1432), where we have located the nucleolin-binding region of WRNp. Our EMSA results indicate that both WRNp and NCL bind to G4 DNA, and that the RGG fragment of nucleolin shows a high affinity to G4 DNA. Indeed, increasing RGG amounts seem to reduce WRNp bound to G4 DNA, while increasing amounts of nucleolin does not seem to have that effect. As nucleolin appears to utilize its RGG region in order to bind both G4 DNA and WRNp, the nucleolin-G4 DNA interaction is perhaps stronger than the NCL-WRNp interaction, and that is why nucleolin is unable to dislodge WRNp as can RGG. These observations indicate a possible competitive mechanism for the regulation of WRNp function by nucleolin: NCL binding to the WRN nucleolar binding region might prevent WRN helicase activity; subsequently, when the NCL-WRN complex is dissociated, for example, by NCL binding to a preferred substrate such as G4 DNA, WRN helicase function is released from inhibition.

What sense can we make of the nucleolar to nucleoplasm trafficking of these proteins in response to genomic stress? The nucleolus was found to contain over 500 proteins [63], many of which are unconnected to nucleic acid metabolism or processing [9]. In response to several types of stress, the nucleolus is depopulated of proteins, and there is a sharp increase in the amount of these proteins in the nucleoplasm, as we have noted for WRNp and NCL. We have previously suggested that the nucleolus serves as a convenient depot for many proteins involved in the response to DNA damage [34], [64]. DNA damage activates these quiescent proteins, perhaps via phosphorylation, and the nucleolar complexes are rapidly disassociated, perhaps by VCP in an ATP dependent process. In support of this scenario we note that both WRNp and VCP are tyrosine phosphorylated in the nucleolus after hydrogen peroxide treatment [26], [65].

Rapid nucleolar protein complex dissociation after DNA damage from CPT, for example, and dispersal to the nucleoplasm, enables DNA damage response proteins such as WRNp and nucleolin to greatly and rapidly increase in the nucleoplasm, where they can be engaged in the formation of DNA repair foci in the proper hierarchical sequence. We propose that nucleolin may inhibit WRNp action in the nucleoplasm until it is required in the DNA repair event. Thus, we find new WRNp-nucleolin complexes (Figure 4 and Figure S2) or WRNp-topoisomerase complexes in the nucleoplasm [51]. The relocation mechanism is a specific response to CPT-induced stress. For example, in cells treated with actinomycin D, both NCL (C23) and Nucleophosmin (B23) remain in the nucleolus, while DNA helicase II left the nucleolus [66], [67]. Therefore, the specific effects on WRN and NCL caused by CPT induced damage (DNA breaks), in which NCL and WRN translocate from the nucleolus to the nucleoplasm and subsequently interact, is neither seen after transcriptional perturbation (Actinomycin D), nor after inhibition of DNA synthesis (HU). We have shown three lines of evidence that the WRN helicase and nucleolin interact: reciprocal immunoprecipitation, immunofluorescent co-localization and *in vitro* binding of purified proteins. Furthermore, our results indicate that this interaction is probably via their C-termini. In addition, we have examined the possible role of NCL on WRN function utilizing WRN in vitro functional assays. We show that NCL can inhibit the WRN helicase, but not exonuclease function, and that they may co-regulate G4 DNA unwinding in the response to certain DNA damaging agents such as CPT. We have postulated that the NCL-WRNp complex may be the inactive form of WRNp that is released from the nucleolus. After increasing in the nucleoplasm and reaching a critical mass, WRNp is released from inhibition and can participate in the DNA repair processes at the required time and sequence. The precise specificity and timing of this response to DNA damage will be the focus of our future research.

## Supporting Information

Figure S1 **WRNp and NCL reciprocally co-immunoprecipitate.** Nuclear extracts (A) or whole cell lysates (B) were immunoprecipitated and immunoblotted as described in Materials and Methods. **A.** Equal amounts of TERT-1604 nuclear extract were immunoprecipitated with anti-NCL mAb (lanes 1 and 7- CalBiochem), or goat anti-WRN (lanes 2 and 8). Rabbit anti-WRN (lanes 1 4) or rabbit anti-NCL (lanes 5 9) were used to detect precipitated proteins and blots were visualized by enhanced chemiluminescence. Control Rabbit IgG (rb IgG, Sigma) precipitates are shown in lanes 4 and 9. Lys-Nuclear pellet proteins extracted by Triton X-100 solubilization (lanes 3 and 5); Pellet-Triton ×-100 insoluble fraction (lane 6). MW in kDa are indicated at left. **B.** Cells as indicated were solubilized with Nonidet NP-40 and equal amounts of lysates were immunoprecipitated with rabbit anti-WRN. Mouse anti-WRN (top) or mouse anti-NCL (bottom) were used to detect precipitated proteins and blots were visualized by enhanced chemiluminescence. AG11395 is a Werner Syndrome cell line that contains abnormal WRNp, which is not precipitated by the anti-WRN. WB- Western blot; IP- immunoprecipitation.
(TIF)

Figure S2 **GFP-NCL and RFP-WRN co-localize in the nucleoplasm after 1.0 **µM **CPT treatment.** U2OS cells were transfected with GFP-NCL (green) and RFP-WRN (red) as described in Materials and Methods. Cells were treated with 1.0 µM CPT and immediately imaged in a time series obtained with a Zeiss 710 confocal. Still images from a 120 minute time series at 0, 3, 10 and 114 minutes after the addition of CPT. An enlarged image of the same nucleoplasmic foci is shown below each frame, illustrating the dynamic nature of the interaction.
(TIF)

Table S1 U2OS cells were transfected as described in Materials and Methods and treated with either 1.0 or 15.0 µM CPT, and images were collected as time series of a single field of cells or multiple fields using the Tile function of the Zeiss Zen software. Double-transfected cells were examined for the appearance of nuclear foci containing both NCL (green) and WRN (red). These cells were counted as “Nuclear Foci Coloc.”, in which foci are non-nucleolar foci in which co-localization was observed in CPT-treated nuclei.
(DOC)

Movie S1 **GFP-NCL and RFP-WRN co-localize in the nucleoplasm after CPT treatment.** A movie of the 240 minute time series of images captured after addition of 15 µM CPT to U2OS cells that were previously transfected with NCL and WRN. Time series was converted with Zen software (Zeiss) to AVI format at 6 fps. Each frame represents 2 minutes. The still images in Figure 4A and 4C were taken from this series.
(AVI)
