# Supplementary figures and images for: Nucleolin Inhibits G4 Oligonucleotide Unwinding by Werner Helicase
Source: PLoS One. 2012 Jun 4;7(6):e35229. doi: 10.1371/journal.pone.0035229 (PMC3366963; doi:10.1371/journal.pone.0035229)

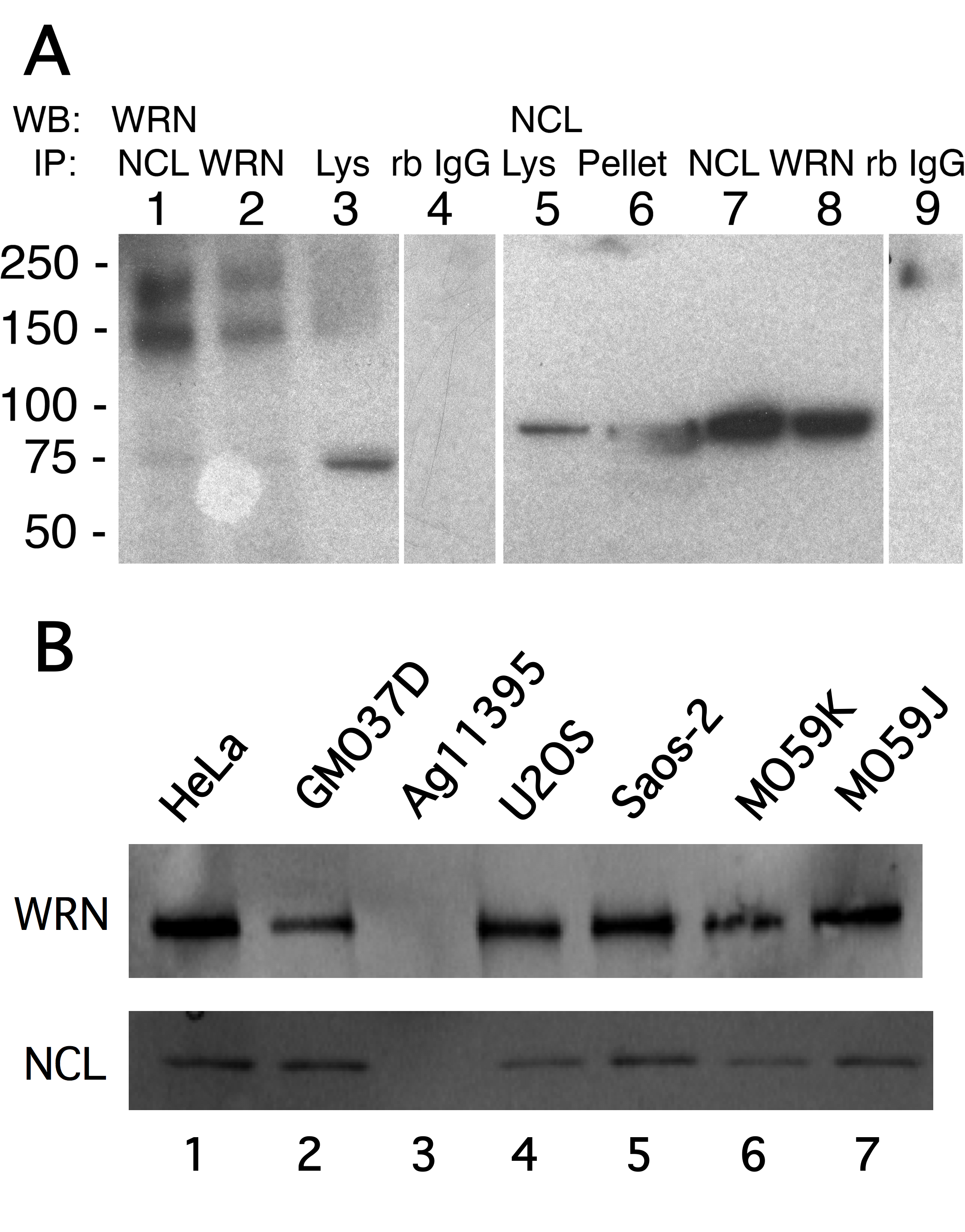

Supplement: Figure S1 — WRNp and NCL reciprocally co-immunoprecipitate. Nuclear extracts (A) or whole cell lysates (B) were immunoprecipitated and immunoblotted as described in Materials and Methods. A. Equal amounts of TERT-1604 nuclear extract were immunoprecipitated with anti-NCL mAb (lanes 1 and 7- CalBiochem), or goat anti-WRN (lanes 2 and 8). Rabbit anti-WRN (lanes 1 4) or rabbit anti-NCL (lanes 5 9) were used to detect precipitated proteins and blots were visualized by enhanced chemiluminescence. Control Rabbit IgG (rb IgG, Sigma) precipitates are shown in lanes 4 and 9. Lys-Nuclear pellet proteins extracted by Triton X-100 solubilization (lanes 3 and 5); Pellet-Triton ×-100 insoluble fraction (lane 6). MW in kDa are indicated at left. B. Cells as indicated were solubilized with Nonidet NP-40 and equal amounts of lysates were immunoprecipitated with rabbit anti-WRN. Mouse anti-WRN (top) or mouse anti-NCL (bottom) were used to detect precipitated proteins and blots were visualized by enhanced chemiluminescence. AG11395 is a Werner Syndrome cell line that contains abnormal WRNp, which is not precipitated by the anti-WRN. WB- Western blot; IP- immunoprecipitation. (TIF) [file pone.0035229.s001.tif]

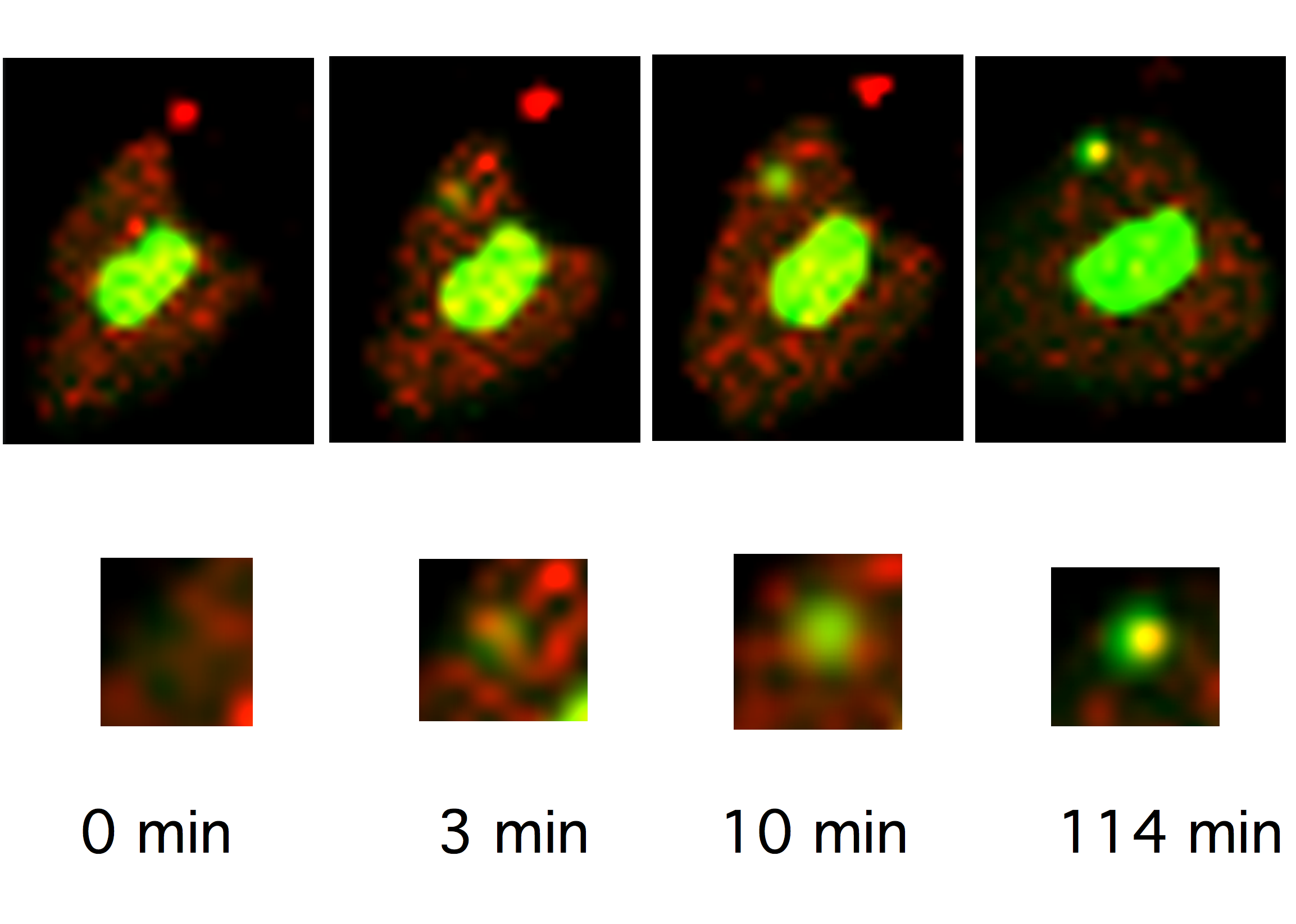

Supplement: Figure S2 — GFP-NCL and RFP-WRN co-localize in the nucleoplasm after 1.0 µM CPT treatment. U2OS cells were transfected with GFP-NCL (green) and RFP-WRN (red) as described in Materials and Methods. Cells were treated with 1.0 µM CPT and immediately imaged in a time series obtained with a Zeiss 710 confocal. Still images from a 120 minute time series at 0, 3, 10 and 114 minutes after the addition of CPT. An enlarged image of the same nucleoplasmic foci is shown below each frame, illustrating the dynamic nature of the interaction. (TIF) [file pone.0035229.s002.tif]
