# Supplementary material for: Nucleolin Inhibits G4 Oligonucleotide Unwinding by Werner Helicase
Source: PLoS One. 2012 Jun 4;7(6):e35229. doi: 10.1371/journal.pone.0035229 (PMC3366963; doi:10.1371/journal.pone.0035229)
Supplement: Table S1 — U2OS cells were transfected as described in Materials and Methods and treated with either 1.0 or 15.0 µM CPT, and images were collected as time series of a single field of cells or multiple fields using the Tile function of the Zeiss Zen software. Double-transfected cells were examined for the appearance of nuclear foci containing both NCL (green) and WRN (red). These cells were counted as “Nuclear Foci Coloc.”, in which foci are non-nucleolar foci in which co-localization was observed in CPT-treated nuclei. (DOC) [file pone.0035229.s003.doc]

A- Transfected U2OS cells treated with 1.0 µM CPT

| Exp. | Total Cells | GFP-NCL plus | RFP-WRN plus | Nucleolar Coloc. | Nuclear Foci Coloc. | % Foci |
| --- | --- | --- | --- | --- | --- | --- |
| 1 | 124 | 22 | 15 | 13 | 7 | 47 |
| 2 | 350 | 31 | 20 | 17 | 10 | 50 |
| 3 | 82 | 7 | 3 | 3 | 1 | 33 |

B- Transfected U2OS cells treated with 15.0 µM CPT

|  |  |  |  |  |  |  |
| --- | --- | --- | --- | --- | --- | --- |
|  |  |  |  |  |  |  |
|  |  |  |  |  |  |  |

| Exp. | Total Cells | GFP-NCL plus cells | RFP-WRN plus cells | Nucleolar Coloc. | Nuclear Foci Coloc. | % Foci |
| --- | --- | --- | --- | --- | --- | --- |
| 1 | 160 | 8 | 5 | 5 | 3 | 60 |
| 2 | 94 | 10 | 6 | 5 | 4 | 67 |
| 3 | 390 | 44 | 18 | 16 | 9 | 50 |
| 4 | 327 | 111 | 54 | 51 | 41 | 76 |
